# Diagnostic Accuracy of Bedside Lung Ultrasound in Detecting Traumatic Pneumothorax by Novice Physicians in the Emergency Department of a Tertiary Care Hospital of Nepal

**DOI:** 10.1155/2024/9956637

**Published:** 2024-09-19

**Authors:** Monisma Malla, Anmol Purna Shrestha, Shailesh Prasad Shrestha, Roshana Shrestha

**Affiliations:** Department of General Practice and Emergency Medicine Kathmandu University School of Medical Sciences, Dhulikhel, Kavrepalanchowk, Bagmati Province, Nepal

## Abstract

**Introduction:**

Traumatic pneumothorax is a life-threatening condition requiring vigilant clinical assessment and urgent management. Lung ultrasound (LUS) is considered to be a safer, rapid, and accurate modality for the early diagnosis of traumatic pneumothorax. The principle objective of this study was to evaluate the diagnostic accuracy of bedside LUS performed by trained novice physicians in the diagnosis of traumatic pneumothorax as compared to supine chest X-rays (CXRs) and/or computed tomography (CT) scans and/or air leak during needle/tube thoracostomy as composite standard.

**Methods:**

It is a prospective, cross-sectional, single-blinded study using a nonprobability quota sampling technique. A total of 96 patients presenting to the emergency department (ED) with polytrauma and chest injuries within a period of twelve months were included. The diagnostic accuracy of bedside LUS performed by trained novice physicians was calculated in terms of sensitivity, specificity, positive predictive value (PPV), and negative predictive value (NPV) and compared with the composite standard.

**Results:**

The sensitivity of LUS in diagnosing traumatic pneumothorax as compared to the composite standard was 100% (95% confidence interval (CI): 59.05%–100.00%), whereas its specificity was 97.75% (95% CI: 92.12%–99.73%). Similarly, the PPV and NPV of LUS were 77.7% (95% CI: 39.99%–97.19%) and 100% (95% CI: 95.85%–100.00%), respectively.

**Conclusion:**

The results of the study showed that the application of LUS in detecting traumatic pneumothorax had similar diagnostic accuracy as supine CXR. Bedside LUS is widely available, portable, and inexpensive. It also has the capability of real-time imaging and can be repeated as necessary with less risk of radiation exposure. Therefore, physicians working in tertiary and rural health institutions must be trained adequately in order to uplift the clinical utility of LUS for the timely and cost-effective detection of traumatic pneumothorax.

## 1. Introduction

Traumatic pneumothorax, the most common life-threatening outcome in chest trauma, occurs in over 20% of patients with blunt injuries and about 40% with penetrating chest injuries [1]. Failure to detect and treat pneumothorax could lead to acute complications including hypoxia, tension pneumothorax, cardiopulmonary failure, or death. LUS is a safer option and has an important diagnostic role in the early detection of clinically significant traumatic pneumothorax, especially in low-resource setting countries [2].

The use of LUS to diagnose pneumothorax was first described in a veterinary medical journal in 1986. The following year, Husain et al. described LUS recognition of pneumothorax in human subjects on the basis of the absence of pleural sliding and comet tail artifacts [3]. The risks of clinical deterioration associated with missing pneumothorax highlight the clinical importance of a safer, more rapid, and more accurate method of diagnosing traumatic pneumothorax [4].

Although the specificity of supine CXR and LUS is similar, the sensitivity of supine CXR is less as compared to LUS in the detection of traumatic pneumothorax [5–8]. Supine CXR requires time, resources, and equipment which may further delay the diagnosis and management of pneumothorax. Also, patients have an increased risk of radiation exposure [9]. Therefore, the entire process of performing supine CXR in trauma patients can be disruptive and may delay resuscitation of critically ill patients [10].

CT scans are considered as the standard criterion, however, in low-resource settings, they require transporting patients out of the resuscitation area and into the diagnostic imaging department which has its inherent risks due to the lack of equipment, space, and personnel to help with resuscitation in case the patients deteriorate. Moreover, patients are exposed to harmful ionizing radiation and financial burdens due to its higher cost.

LUS has numerous advantages when compared to supine CXR and CT scans, which include nonusage of ionizing radiations, being portable, capable of real-time imaging, and suited for repeat examinations as necessary [11]. It is widely available and inexpensive. Therefore, LUS training for novice physicians especially in low-resource settings could lead to timely diagnosis and treatment, thereby reducing pneumothorax-related complications and improving patient outcomes. Various studies have shown LUS to have higher accuracy in detecting traumatic pneumothorax with short, structured training for novice physicians [12–15].

The main objective of this study was to assess the diagnostic accuracy of bedside LUS performed by novice physicians in detecting traumatic pneumothorax following short training as compared to standardized methods in the ED. The specific objectives were to determine the various findings of bedside LUS in pneumothorax and to compare the diagnostic accuracy of bedside LUS performed by trained novice physicians with either supine CXR and/or CT scans and/or air leak during tube/needle thoracostomy as the composite standard. Moreover, we also aimed to compare the diagnostic accuracy of LUS and supine CXR with CT scans and/or air leaks during tube/needle thoracostomy as the gold standard.

## 2. Materials and Methods

### 2.1. Study Design

We conducted a prospective, cross-sectional, single-blinded study carried out within a period of twelve months from 20^th^ October, 2021 to 18^th^ October, 2022. We performed nonprobability quota sampling in order to enroll an equal number of patients presenting from all three shifts at the ED of Dhulikhel Hospital.

### 2.2. Study Setting

Dhulikhel Hospital is a community-based teaching hospital that has been serving patients from more than 50 out of 75 districts of the country. It is a tertiary-level hospital with a 30-bed ED which has approximately 20,000 patient visits annually and has a high acuity level as per internal audits. The department is run under the supervision of five consultant emergency physicians, 10 medical officers, and 40 nurses and paramedics.

### 2.3. Study Population

The patients who presented to the ED with polytrauma; suspected traumatic chest injuries; whose LUS was performed by trained novice physicians; either supine CXR, CT chest, or tube thoracostomy performed; and patients giving written consent (or caretakers in cases of children <16 years and patients >16 years unable to give consent) were included in the study. The patients who presented with open chest injuries, subcutaneous emphysema, chest tube insertion or needle decompression before LUS by trained novice physicians, tension pneumothorax, and those who denied consent were excluded from the study.

### 2.4. Study Procedure

The novice physicians are posted to the ED for a one-month internship rotation. At the beginning of their clinical rotation, they were trained with a short course on extended focused assessment with sonography in trauma (EFAST). The course consisted of didactic lectures of two hours provided by EFAST-trained faculties with the help of standardized training materials and recorded videos based on the European Federation of Societies for Ultrasound in Medicine and Biology (EFSUMB) [16]. The practice session consisted of two hours duration which included bedside LUS evaluation on at least five normal individuals. Then, the novice physicians were tested for their knowledge and skills through objective structured clinical examination (OSCE) (Annex IV). Following this, they were considered eligible to perform LUS as a part of this study.

On arrival, patients with suspected traumatic chest injuries first underwent regular triaging as a part of ED protocol where they were categorized into different zones on the basis of their clinical status. Then, they were thoroughly assessed and examined, followed by appropriate resuscitation and management. Following the initial stabilization, the trained novice physicians performed EFAST, and the results were recorded. Later if needed the EFAST scan was carried out by senior physicians or medical officers who had been working at the ED for more than six months and were experienced in EFAST scans. In case, the LUS was performed by senior physicians prior to the novice physicians, the novice physicians were blinded to the LUS performed prior by the senior physicians.

**Figure 1:**
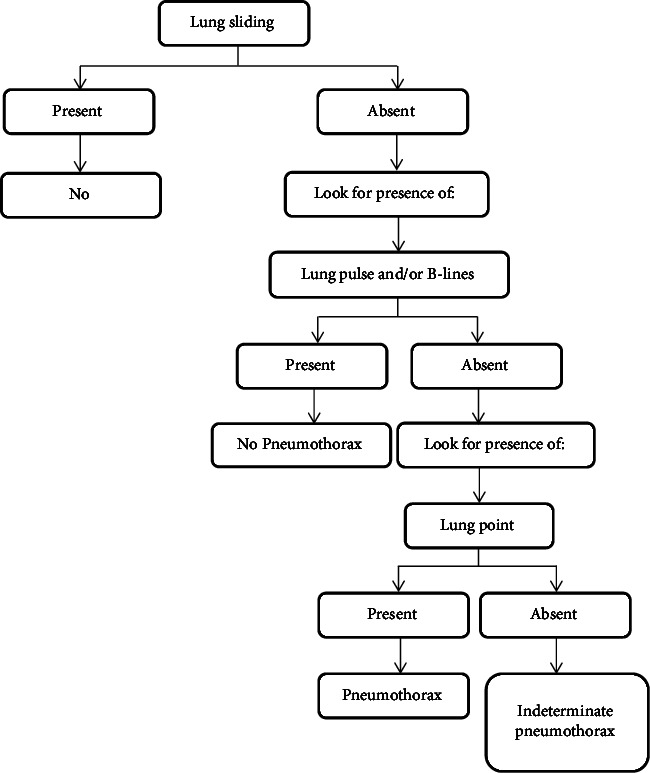
Flowchart illustrating LUS detection of pneumothorax.

LUS was performed by an ultrasound machine (Mindray®) using a curvilinear transducer probe of model P4–2s (2016–10).  Figure 1 shows the algorithm for diagnosing pneumothorax via LUS [17]. First, lung sliding was assessed in the second intercostal space at the midclavicular line. We have made the following corrections in the flowchart.

In this study, if the lung sliding was absent but the lung pulse and/or B-lines were visualized, pneumothorax was ruled out. If the lung sliding sign was absent, the probe was moved to the lateral areas of the thorax to find the lung point sign, which if present was considered to be pneumothorax. If the lung sliding was absent, but there was no lung point, it was considered indeterminate and findings were compared with supine CXR and/or CT scan.

The lung point is considered a 100% specific sign of pneumothorax, but its sensitivity is limited since it cannot be detected in every pneumothorax patient [18]. Therefore, the lung point sign has been taken only as an additional sign in this study. To avoid missing cases by novice physicians, we considered diagnosing pneumothorax in this study based on the absence of lung sliding and the absence of lung pulse and/or B-lines irrespective of the presence or absence of the lung point sign.

As per the clinical stability of the patient, supine CXR, CT scans, or tube/needle thoracostomy were ordered and the results were recorded in the proforma after initial bedside LUS was performed by the novice physicians. The presence of pneumothorax on CT scans and/or supine CXR and/or air leak during needle or tube thoracostomy was taken as the composite standard and was used to compare with LUS findings. A second analysis was performed for those patients who had CT chest and/or tube/needle thoracostomy to constitute a gold standard, allowing calculation of the comparative performance of LUS with supine CXR.

The entire process from the arrival of the patients to initial management and investigations was properly documented in the clinical proforma by the novice physicians. The admitted patients were followed up in their respective departments of admission for one week to follow up on their clinical outcomes.

### 2.5. Sample Size

As the prevalence of chest trauma was unknown, the sample size of the patients in our study was calculated using the standard Cochran's formula taking 10% as the margin of error. Therefore, the sample size required for this study was calculated to be 96.

### 2.6. Statistical Methods

After the data collection was completed, we entered the data into a Microsoft Excel spreadsheet 2010 and analyzed the data using Statistical Package for the Social Sciences (SPSS) software (SPSS Statistics 25).

For descriptive statistics, we used frequency (percentage) and mean ± standard deviation or median with interquartile range for continuous parameters whichever was applicable. Graphical and tabular presentations were also plotted. We analyzed categorical variables by cross-tabulation in two groups using chi-square. Diagnostic tests were estimated using a 2 × 2 table and online MedCalc's diagnostic test evaluator. The accuracy of LUS in identifying pneumothorax was measured by calculating the sensitivity, specificity, PPV, and NPV.

## 3. Results

A total of 96 patients fulfilled the inclusion criteria and were enrolled in our study. All of them underwent bedside LUS by trained novice physicians and supine CXR for the diagnosis of pneumothorax after initial stabilization. A total of 12 CT scans were performed, out of which, three (25%) were positive for pneumothorax as shown in Table 1. The number of patients who underwent tube thoracostomy was six (6.25%), four of which were performed prior to the CT confirmation due to clinical instability. The total number of confirmed positive cases of traumatic pneumothorax was seven (7.3%) considering supine CXR, CT scans, and/or air leak during tube/needle thoracostomy as the composite standard whereas there were nine (9.3%) positive cases of pneumothorax on LUS. The total number of CT scans performed and/or air leaks during tube/needle thoracostomy (*n* = 16) were considered as the gold standard for the diagnosis of traumatic pneumothorax. Out of these, seven (43.75%) patients had pneumothorax.

### 3.1. Baseline Characteristics

The demography and clinical parameters of the study population are listed in Table 2:

### 3.2. Accuracy of LUS in Detection of Pneumothorax

The absence of lung sliding, lung pulse, and/or B-lines with or without the presence of lung point by LUS was considered as the presence of pneumothorax.

#### 3.2.1. Lung Sliding, Lung Pulse, and/or B-Lines

The total number of patients with absent lung sliding, absent lung pulse, and/or B-lines was nine (9.37%). Among these nine patients with absent lung sliding, lung pulse, and/or B-lines, lung point was present in three (33.33%) of the cases.

#### 3.2.2. Lung Point

The total number of patients with the presence of lung points was three (33.33%). In comparison with the composite standard, the specificity and PPV of lung point sign were 100% ((95% CI: 15.81%–100%) and (95% CI: 29.24%–100%)). The sensitivity was 42.86% (95% CI: 9.90%–81.59%) and NPV was 33.33% (95% CI: 20.84%–48.71%) for the presence of pneumothorax.

Table 3 summarizes the diagnostic accuracy of individual LUS findings in comparison with the composite standard.

### 3.3. Overall LUS Performance

**Figure 2:**
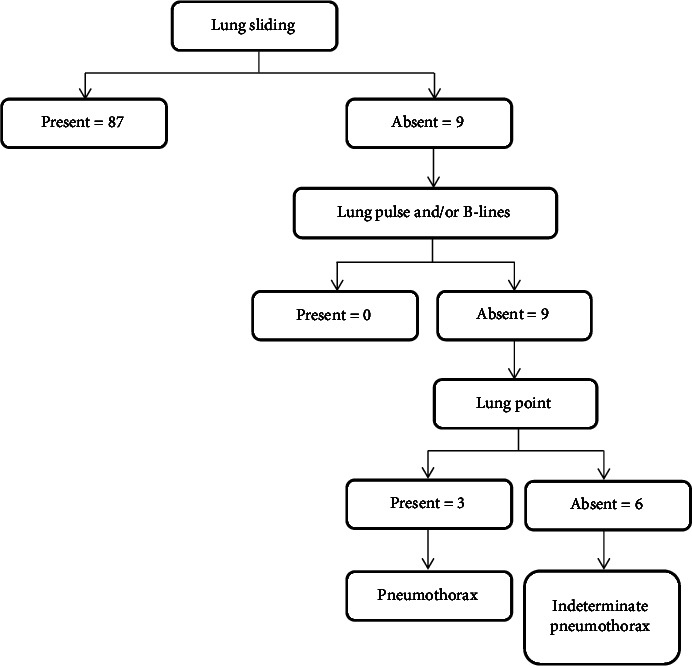
Flowchart illustrating LUS detection of pneumothorax in this study.

This study showed that there was absent lung sliding along with the absence of B-lines and lung pulse in nine (9.3%) cases. Out of the nine cases, lung point was present in three cases. Thus, LUS was overall positive in nine (9.3%) cases and negative in 87 (90.7%) cases as shown in Figure 2.

### 3.4. LUS vs Composite Standard

The diagnostic accuracy of LUS was compared with the composite standard which showed that the sensitivity of LUS was 100% (95% CI: 59.04%–100%), specificity was 97.75% (95% CI: 92.12%–99.73%), PPV was 77.78% (95% CI: 39.99%–97.19%), and NPV was 100% (95% CI: 95.85%–100%). Similarly, the accuracy was 97.92% (95% CI: 92.68%–99.75%) and the positive likelihood ratio was 44.50 (11.30–175.17).

### 3.5. LUS vs Gold Standard

LUS correctly identified seven (43.75%) cases of pneumothorax and falsely inferred one (6.25%) case as compared to the gold standard (*n* = 16). Also, there were eight (50%) true negatives on LUS. The sensitivity of LUS in accurately diagnosing pneumothorax as compared to the gold standard was calculated to be 100% (95% CI: 59.04%–100.00%), whereas its specificity was 88.89% (95% CI: 51.75%–99.72%). Similarly, the PPV and NPV of LUS were 87.5% (95% CI: 47.35%–99.68%) and 100% (95% CI: 63.06%–100.00%), respectively.

### 3.6. CXR Performance

The study showed that the sensitivity and specificity of supine CXR in comparison with the gold standard were 100% (95% CI: 59.04%–100% and 66.37%–100%), respectively. Similarly, PPV and NPV were 100% with a 95% CI of 59.04%–100% and 66.37%–100%, respectively.

Table 4 shows the comparison of LUS with the composite standard, LUS with the gold standard, and supine CXR with the gold standard, respectively.

## 4. Discussion

The present study showed that the diagnostic accuracy of LUS performed by trained novice physicians for the detection of traumatic pneumothorax was higher in terms of sensitivity and specificity. The sensitivity and specificity of LUS in accurately diagnosing pneumothorax as compared to the composite standard were calculated to be 100% and 97.75%, respectively. In addition, the PPV and NPV of LUS were 77.7% and 100%, respectively. The study also showed that the sensitivity, specificity, PPV, and NPV of supine CXR in comparison with composite standard were 100%.

### 4.1. Demographic Variables

In this study, out of the total 96 patients enrolled, 73 (76%) were male and 23 (24%) were female with male to female ratio of 3 : 1. This was similar to the study carried out by Shrestha et al. at Dhulikhel Hospital where most of the trauma patients were male (male: female = 3.8 : 1). The minimum age group in our study was 13 years and the maximum age was 84 years with mean age of 40.05 years. The majority of the cases in our study were within the age group of 21 to 30 years, which was 30 (31.3%). This is the most fruitful age group and belongs to the youths who are responsible for building the future nation. This was similar to the study findings of Chapagain D which had a mean of 40 years with the age ranging from 7 to 87 years [19]. The most common mechanism of injury in our study was found to be RTA, which accounted for 50 (52.08%) cases. This was followed by fall injuries (*n* = 42, 43.75%), physical assault (*n* = 3, 3.1%), and one (1%) patient was buried in the landslide. This was also equivalent to the study by Bhatta et al. where the most common mechanism of trauma was RTA (32.8%), followed by fall injuries (25.4%) and animal/insect-related injuries (20.1%) [20].

### 4.2. Incidence of Pneumothorax

The incidence of pneumothorax was slightly less as compared to the study by Blaivas et al. where pneumothorax was detected in 53 (30%) patients by LUS and 40 (30%) patients by supine CXR out of the total 176 patients who were enrolled in the study over an eight-month period [21]. Most of the serious cases in ED were first attended by medical officers and provided with immediate resuscitation which may have resulted in novice physicians assessing less major trauma cases triaged into red, orange, or yellow categories and therefore, fewer enrollments of pneumothorax cases in the study. On the other hand, the study carried out by Ku et al. which compared LUS with supine CXR, CT chest, or air leak during chest tube insertion showed that 47 of the 549 patients had traumatic pneumothorax, for an incidence of 9% which was comparable with our study [22].

### 4.3. Diagnostic Accuracy of LUS

In this study, the sensitivity and NPV of lung sliding, lung pulse, and B-lines in the detection of pneumothorax were 100% as compared to the composite standard with a 95% CI of 59.04%–100%. Similarly, the specificity and PPV were 97.75% (95% CI: 92.12%–99.73%) and 77.78% (95% CI: 47.07%–93.23%), respectively. Rowan et al. conducted a study which showed that the sensitivity and NPV of LUS were 100%, specificity was 94%, and PPV was 92%. Therefore, the sensitivity and NPV of LUS were comparable with the study by Rowan et al. which was 100% [23]. The specificity and PPV of LUS in our study were less than the study by Rowan et al. The prospective study carried out by Jahanshir et al. showed a sensitivity of LUS of 75%, specificity of 100%, PPV of 100%, and NPV of 94.9% for the diagnosis of pneumothorax. The sensitivity and NPV in our study were higher as compared to their study. However, the specificity and PPV of our study were less compared to the study by Jahanshir et al. [24].

### 4.4. Diagnostic Accuracy of Supine CXR

On comparison with the composite standard, supine CXR had 100% sensitivity (95% CI: 59.04%–100%), 100% specificity (95% CI: 95.94%–100%), 100% PPV (95% CI: 59.04%–100%), and 100% NPV (95% CI: 95.94–100%). This was similar to the evidence-based review of MEDLINE and Embase databases performed by R. Gentry Wilkerson and Michael B. Stone which revealed that the specificity of supine CXR was 100% in all included studies [25]. Also, the sensitivity of supine CXR for the detection of pneumothorax ranged from 28% to 75% in their study. The sensitivity and PPV of supine CXR in this study were equivalent to the study conducted by Abbasi et al., where the specificity and PPV of supine CXR were also 100%. In addition, the sensitivity and NPV of supine CXR in our study were higher as compared to the sensitivity of 48.6% and NPV of 85.1% in their study [26].

### 4.5. Comparison of LUS and Supine CXR

This study showed that the sensitivity and NPV of LUS were 100% (95% CI: 59.04%–100%) as compared to the composite standard and the gold standard, which was equivalent to that of supine CXR. The specificity and PPV of LUS were slightly less as compared to the supine CXR which was 97.75% (95% CI: 92.12%–99.73%) and 77.78% (95% CI: 39.99%–97.19%), respectively. The study carried out in British Columbia by Rowan et al. showed that LUS had a higher sensitivity and NPV, which was 100% with a specificity of 94% and a PPV of 92%, respectively [23]. Supine CXR had 100% specificity and PPV with 36% sensitivity and 70% NPV. The sensitivity and NPV of LUS were higher than supine CXR in both of our studies. However, the specificity and PPV of LUS in our study were less than supine CXR as compared to their study.

The study carried out by Blaivas et al. showed that LUS had a higher sensitivity of 98.1% (95% CI: 89.9%–99.9%) than supine CXR which was 75.5% (95% CI: 61.7%–86.2%) [20]. The specificity of LUS and supine CXR was similar which was 99.2% (95% CI: 95.6%–99.9%) and 100% (95% CI: 97.1%–100%), respectively. Although, the sensitivity of LUS as compared to supine CXR was similar in both of the studies, the specificity was less in our study as compared to their study.

### 4.6. Limitations

The study was carried out in a small sample size within a limited study duration. A convenient sampling method was used, which may result in sampling bias as many samples with traumatic pneumothorax would be missed in between. As a CT scan was not performed in all cases of traumatic chest injuries due to financial constraints of the patients, there were fewer number of sample patients in the gold standard undergoing CT scans. A comparison of the LUS findings performed by trained novice physicians could not be performed with the LUS findings performed by experienced ED team members (medical officers and consultants) after initial resuscitation. Detection of traumatic pneumothorax by LUS is operator-dependent and also depends upon the clinical skills and expertise of the performing novice physicians. The study included patients from the ED of a single tertiary care hospital with the facilities of LUS, CXR, and CT chest, thereby limiting the generalization to other health institutions with limited resources.

## 5. Conclusion

Our study shows that the sensitivity and specificity of LUS in detecting traumatic pneumothorax performed by novice physicians were high, even when performed after a short training. LUS performed by novice physicians had similar diagnostic accuracy as that of supine CXR. Although various studies have shown LUS to be more sensitive and specific than supine CXR for diagnosing traumatic pneumothorax, it is highly user-dependent and depends upon the level of clinical skills and expertise of the performing novice physician. Physical examination, on the other hand, may be inaccurate in identifying significant pathologies in thoracic trauma in hectic ED scenarios. LUS training will provide novice physicians with abundant clinical skills and knowledge in the timely detection of pneumothorax, thereby reducing pneumothorax-related life-threatening conditions. Thus, novice physicians must be encouraged to routinely utilize LUS in assessing trauma cases for detection of pneumothorax and provide early interventions as necessary.

## Figures and Tables

**Table 1:** Composite standard (supine CXR + CT scan + tube/needle thoracostomy).

Composite standard	Total performed	Positive cases		Negative cases	
Supine CXR	96 (100%)	7 out of 96	7.3%	89 out of 96	92.7%
CT scan	12 (12.5%)	3 out of 12	25%	9 out of 12	75%
Tube/needle thoracostomy	6 (6.25%)	6 out of 6	100%	0 out of 6	0.0%

CXR, chest X-ray; CT, computed tomography.

**Table 2:** Clinical parameters of the study population, *n* = 96.

S. no	Baseline characteristics	Number of patients	Percentage
1	Age category (years)		
	10–20	8	8.3
	21–30	30	31.3
	31–40	11	11.5
	41–50	12	12.5
	51–60	21	21.9
	61–70	4	4.2
	71–80	9	9.3
	81–90	1	1.0

2	Gender		
	Male	73	76
	Female	23	24

3	Timing of presentation		
	Morning	32	33.4
	Evening	34	35.4
	Night	30	31.2

4	Triage category		
	Red	19	19.7
	Orange	8	8.3
	Yellow	48	50
	Green	21	21.8

5	Mode of injury		
	Road traffic accident (RTA)	50	52.08
	Fall injury	42	43.75
	Physical assault	3	3.1
	Buried in landslide	1	1

6	Mode of transport		
	Private vehicle	60	62
	Ambulance	33	34
	Taxi	2	2
	Police van	1	1
	Walking	1	1

7	Disposition from ED		
	Discharged	60	62
	Admitted department	36	38
	CTVS	7	19.5
	Surgery	7	19.5
	Neurosurgery	8	22.2
	Orthopedics	13	36
	Medicine	1	2.8
	Referral	2	2

RTA, road traffic accident; ED, emergency department; CTVS, cardiothoracic and vascular surgery.

**Table 3:** Diagnostic accuracy of individual LUS features on comparison with composite standard.

LUS findings	LUS vs. composite standard			
	Sensitivity	Specificity	PPV	NPV
Lung sliding	100% (95% CI: 59.04%–100%)	97.75% (95% CI: 92.12%–99.73%)	77.78% (95% CI: 47.07%–93.23%)	100% (95% CI: 95.85%–100%)
B-lines	100% (95% CI: 59.04%–100%)	97.75% (95% CI: 92.12%–99.73%)	77.78% (95% CI: 47.07%–93.23%)	100% (95% CI: 95.85%–100%)
Lung pulse	100% (95% CI: 59.04%–100%)	97.75% (95% CI: 92.12%–99.73%)	77.78% (95% CI: 47.07%–93.23%)	100% (95% CI: 95.85%–100%)
Lung point	42.86% (95% CI: 9.90%–81.59%)	100% (95% CI: 15.81%–100%)	100% (95% CI: 29.24%–100%)	33.33% (95% CI: 20.84%–48.71%)

LUS, lung ultrasound; CI, confidence interval; PPV, positive predictive value; NPV, negative predictive value.

**Table 4:** Diagnostic test performances in the detection of posttraumatic pneumothorax.

	LUS vs. composite standard (*n* = 96)		LUS vs. gold standard (*n* = 16)		CXR vs. gold standard (*n* = 16)	
	%	95% CI	%	95% CI	%	95% CI
Sensitivity	100	59.04–100	100	59.04–100	100	59.04–100
Specificity	97.75	92.12–99.73	88.89	51.75–99.72	100	66.37–100
PPV	77.78	39.99–97.19	87.50	47.35–99.68	100	59.04–100
NPV	100	95.85–100	100	63.06–100	100	66.37–100
Accuracy	97.92	92.68–99.75	93.75	69.77–99.84	100	79.41–100
LR of positive test	44.5	11.30–175.15	9.00	1.42–57.12		
LR of negative test	0.00		0.00		0.00	

LUS, lung ultrasound; CXR, chest X-ray; CI, confidence interval; PPV, positive predictive value; NPV, negative predictive value; LR, likelihood ratio.

## Data Availability

The data used to support the findings of this study are included within the article.

## Supplementary Materials

Supplementary Materials Annex I: training protocol in LUS to detect pneumothorax for novice physicians.

## References

[1] Mumtaz U., Zahur Z., Chaudhry M. A., Warraich R. A. (2016). Bedside ultrasonography: a useful tool for traumatic pneumothorax. *Journal of College of Physicians and Surgeons Pakistan*.

[2] Volpicelli G., Elbarbary M., Blaivas M. (2012). International evidence-based recommendations for point-of-care lung ultrasound. *Intensive Care Medicine*.

[3] Husain L. F., Hagopian L., Wayman D., Baker W. E., Carmody K. A. (2012). Sonographic diagnosis of pneumothorax. *Journal of Emergencies, Trauma, and Shock*.

[4] Filosso P. L., Guerrera F., Sandri A. (2017). Errors and complications in chest tube placement. *Thoracic Surgery Clinics*.

[5] Bridwell R. E., Long B., Gottlieb M. (2021). Is chest ultrasonography superior to supine chest radiography in identifying pneumothorax in emergency department trauma patients?. *Annals of Emergency Medicine*.

[6] Abdalla W., Elgendy M., Abdelaziz A. A., Ammar M. A. (2016). Lung ultrasound versus chest radiography for the diagnosis of pneumothorax in critically ill patients: a prospective, single-blind study. *Saudi Journal of Anaesthesia*.

[7] Chan K. K., Joo D. A., McRae A. D. (2020). Chest ultrasonography versus supine chest radiography for diagnosis of pneumothorax in trauma patients in the emergency department. *Cochrane Database of Systematic Reviews*.

[8] Ebrahimi A., Yousefifard M., Mohammad Kazemi H. (2014). Diagnostic accuracy of chest ultrasonography versus chest radiography for identification of pneumothorax: a systematic review and meta-analysis. *Tanaffos*.

[9] Stojanovska J., Hurwitz Koweek L. M., Chung J. H. (2020). ACR appropriateness Criteria® blunt chest trauma-suspected cardiac injury. *Journal of the American College of Radiology*.

[10] Noppen M., De Keukeleire T. (2008). Pneumothorax. *Respiration*.

[11] Kaur J., Bhoil R., Kumar R., Attri P. K., Thakur R. (2021). Diagnosis of traumatic pneumothorax: a comparison between lung ultrasound and supine chest radiographs. *Indian Journal of Critical Care Medicine*.

[12] Fissore E., Zieleskiewicz L., Markarian T. (2021). Pneumothorax diagnosis with lung sliding quantification by speckle tracking: a prospective multicentric observational study. *The American Journal of Emergency Medicine*.

[13] Cazes N., Desmots F., Geffroy Y., Renard A., Leyral J., Chaumoître K. (2013). Emergency ultrasound: a prospective study on sufficient adequate training for military doctors. *Diagnostic and Interventional Imaging*.

[14] Basnet S., Shrestha S. K., Pradhan A. (2020). Diagnostic performance of the extended focused assessment with sonography for trauma (EFAST) patients in a tertiary care hospital of Nepal. *Journal of Trauma and Acute Care Surgery*.

[15] House D. R., Amatya Y., Nti B., Russell F. M. (2021). Lung ultrasound training and evaluation for proficiency among physicians in a low-resource setting. *The Ultrasound Journal*.

[16] Cantisani V., Dietrich C. F., Badea R. (2016). EFSUMB statement on medical student education in ultrasound [long version]. *Ultrasound International Open*.

[17] Lichtenstein D. A. (2014). Lung ultrasound in the critically ill. *Current Opinion in Critical Care*.

[18] Chen L., Zhang Z. (2015). Bedside ultrasonography for diagnosis of pneumothorax. *Quantitative Imaging in Medicine and Surgery*.

[19] Chapagain D., Reddy D. J., Shah S., Shrestha K. G. (2015). Diagnostic modalities x-ray and CT chest differ in the management of thoracic injury. *Journal of College of Medical Sciences-Nepal*.

[20] Bhatta S., Magnus D., Mytton J. (2021). The epidemiology of injuries in adults in Nepal: findings from a hospital-based injury surveillance study. *International Journal of Environmental Research and Public Health*.

[21] Blaivas M., Lyon M., Duggal S. (2005). A prospective comparison of supine chest radiography and bedside ultrasound for the diagnosis of traumatic pneumothorax. *Academic Emergency Medicine*.

[22] Ku B. S., Fields J. M., Carr B., Everett W. W., Gracias V. H., Dean A. J. (2013). Clinician-performed bedside ultrasound for the diagnosis of traumatic pneumothorax. *Western Journal of Emergency Medicine*.

[23] Rowan K. R., Kirkpatrick A. W., Liu D., Forkheim K. E., Mayo J. R., Nicolaou S. (2002). Traumatic pneumothorax detection with thoracic US: correlation with chest radiography and CT-initial experience. *Radiology*.

[24] Jahanshir A., Moghari S. M., Ahmadi A., Moghadam P. Z., Bahreini M. (2020). Value of point-of-care ultrasonography compared with computed tomography scan in detecting potential life-threatening conditions in blunt chest trauma patients. *Ultrasound J*.

[25] Wilkerson R. G., Stone M. B. (2010). Sensitivity of bedside ultrasound and supine anteroposterior chest radiographs for the identification of pneumothorax after blunt trauma. *Academic Emergency Medicine*.

[26] Abbasi S., Farsi D., Hafezimoghadam P., Fathi M., Zare M. A. (2013). Accuracy of emergency physician-performed ultrasound in detecting traumatic pneumothorax after a 2-h training course. *European Journal of Emergency Medicine*.

