# Supplementary material for: Diagnostic Accuracy of Bedside Lung Ultrasound in Detecting Traumatic Pneumothorax by Novice Physicians in the Emergency Department of a Tertiary Care Hospital of Nepal
Source: Emerg Med Int. 2024 Sep 19;2024:9956637. doi: 10.1155/2024/9956637 (PMC11427713; doi:10.1155/2024/9956637)
Supplement: Supplementary Materials — Annex I: training protocol in LUS to detect pneumothorax for novice physicians. [file 9956637.f1.pdf]

Annex I: Training protocol in LUS to detect pneumothorax for novice physicians

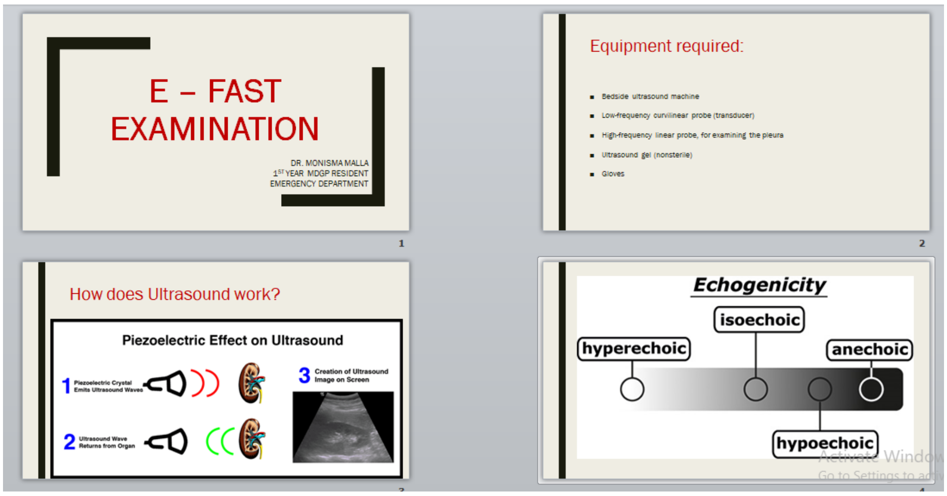

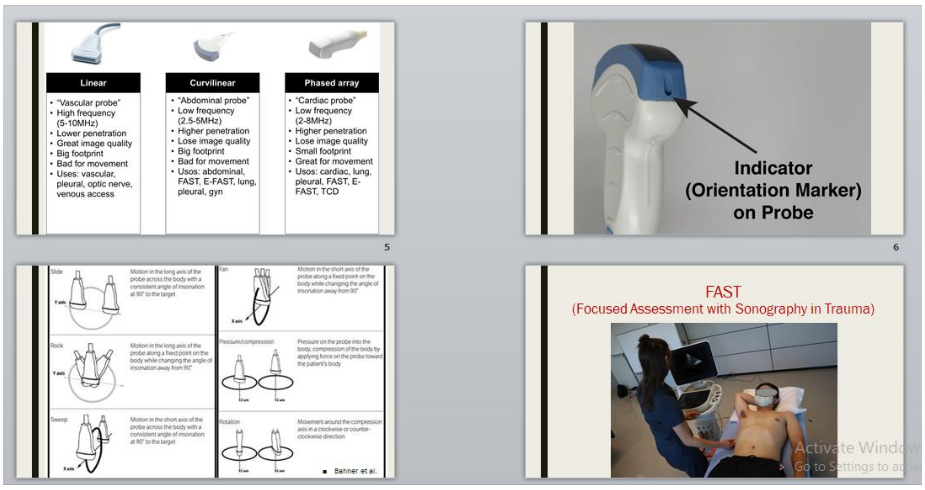

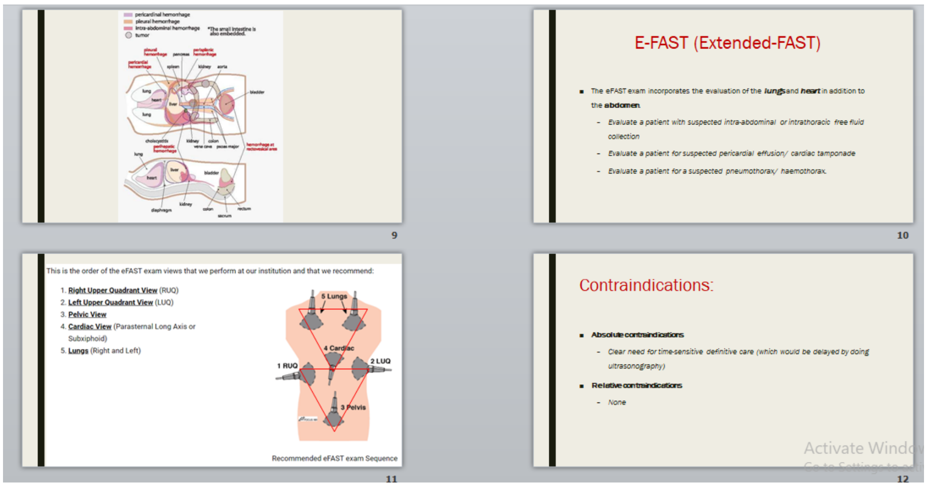

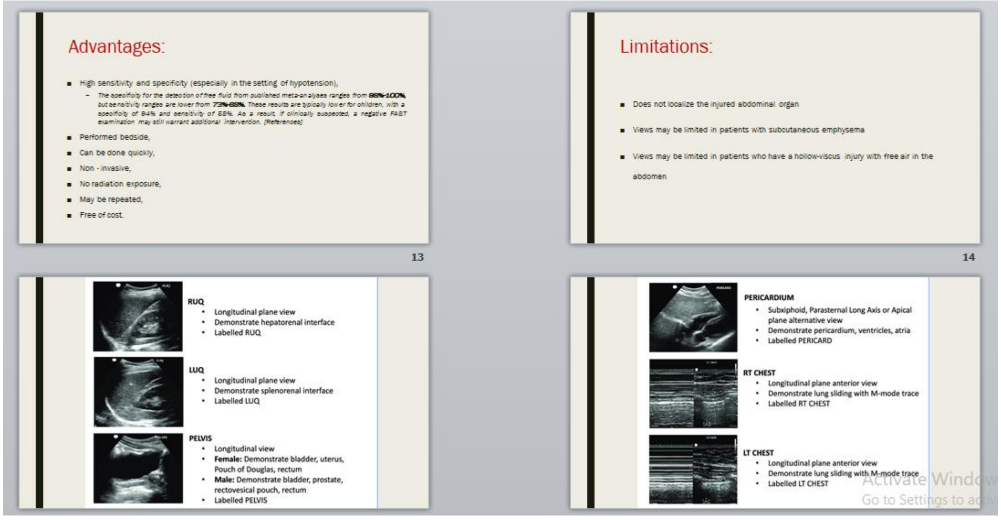

Advantages:
High sensitivity and specificity (especially in the setting of hypotension),
Performed bedside,
Can be done quickly,
Non - invasive,
No radiation exposure,
May be repeated,
Free of cost.
13
Limitations:
Does not localize the injured abdominal organ
Views may be limited in patients with subcutaneous emphysema
Views may be limited in patients who have a hollow-viscus injury with free air in the abdomen
14
RUQ
Longitudinal plane view
Demonstrate hepatorenal interface
Labelled RUQ
LUQ
Longitudinal plane view
Demonstrate splenorenal interface
Labelled LUQ
PELVIS
Longitudinal view
Female: Demonstrate bladder, uterus, Pouch of Douglas, rectum
Male: Demonstrate bladder, prostate, rectovesical pouch, rectum
Labelled PELVIS
PERICARDIUM
Subxiphoid, Parasternal Long Axis or Apical plane alternative view
Demonstrate pericardium, ventricles, atria
Labelled PERICARD
RT CHEST
Longitudinal plane anterior view
Demonstrate lung sliding with M-mode trace
Labelled RT CHEST
LT CHEST
Longitudinal plane anterior view
Demonstrate lung sliding with M-mode trace
Labelled LT CHEST

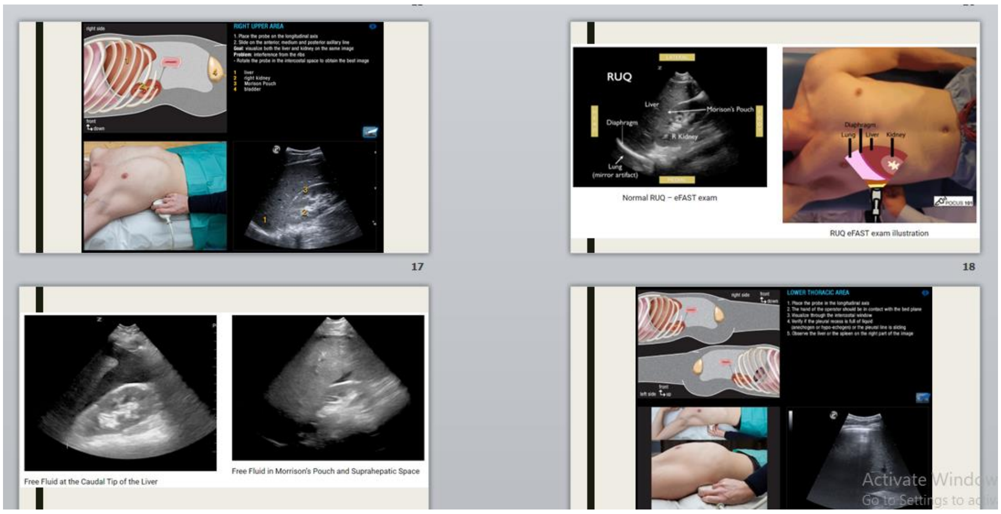

RIGHT UPPER AREA
17
RUQ
Liver
Morison's Pouch
Diaphragm
R Kidney
Lung (mirror artifact)
Normal RUQ – eFAST exam
Diaphragm
Lung
Liver
Kidney
RUQ eFAST exam illustration
18
Free Fluid at the Caudal Tip of the Liver
Free Fluid in Morrison's Pouch and Suprahepatic Space
LOWER THORACIC AREA

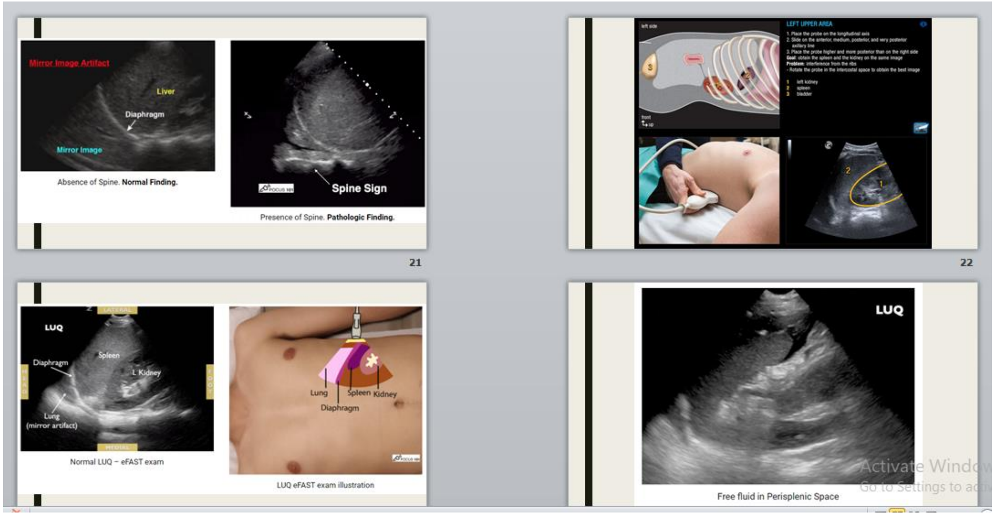

Mirror Image Artifact
Liver
Diaphragm
Mirror Image
Absence of Spine. Normal Finding.
Spine Sign
Presence of Spine. Pathologic Finding.
21
LEFT UPPER AREA
22
LUQ
Diaphragm
Spleen
L Kidney
Lung (mirror artifact)
Normal LUQ – eFAST exam
Lung
Spleen
Kidney
Diaphragm
LUQ eFAST exam illustration
LUQ
Free fluid in Perisplenic Space

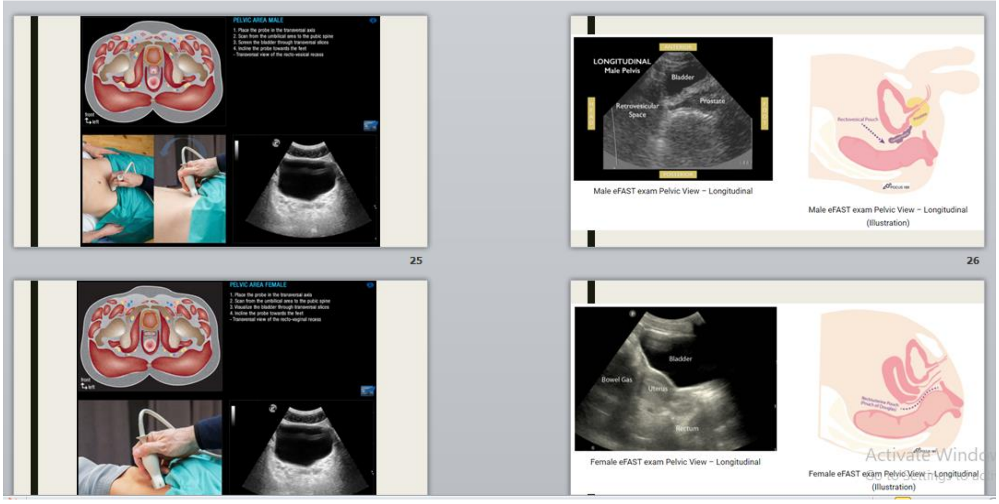
PELVIC AREA MALE
LONGITUDINAL
Male Pelvis
Bladder
Retrovesicular Space
Prostate
Male eFAST exam Pelvic View – Longitudinal
Male eFAST exam Pelvic View – Longitudinal (Illustration)
25
26
PELVIC AREA FEMALE
Bladder
Bowel Gas
Uterus
Rectum
Female eFAST exam Pelvic View – Longitudinal
Female eFAST exam Pelvic View – Longitudinal (Illustration)

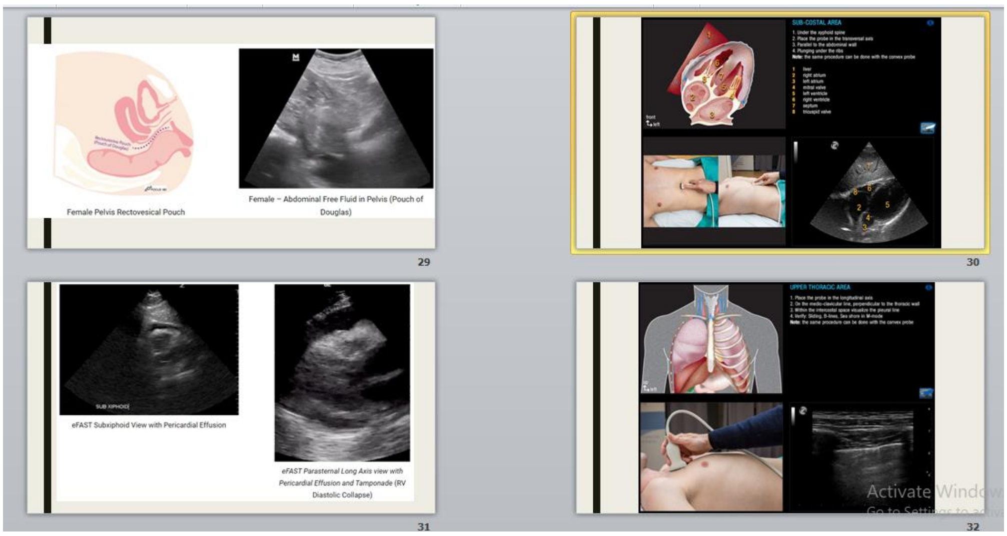
Female Pelvis Rectovesical Pouch
Female – Abdominal Free Fluid in Pelvis (Pouch of Douglas)
29
SUB-COSTAL AREA
30
SUB XIPHOID
eFAST Subxiphoid View with Pericardial Effusion
eFAST Parasternal Long Axis view with Pericardial Effusion and Tamponade (RV Diastolic Collapse)
31
UPPER THORACIC AREA
32

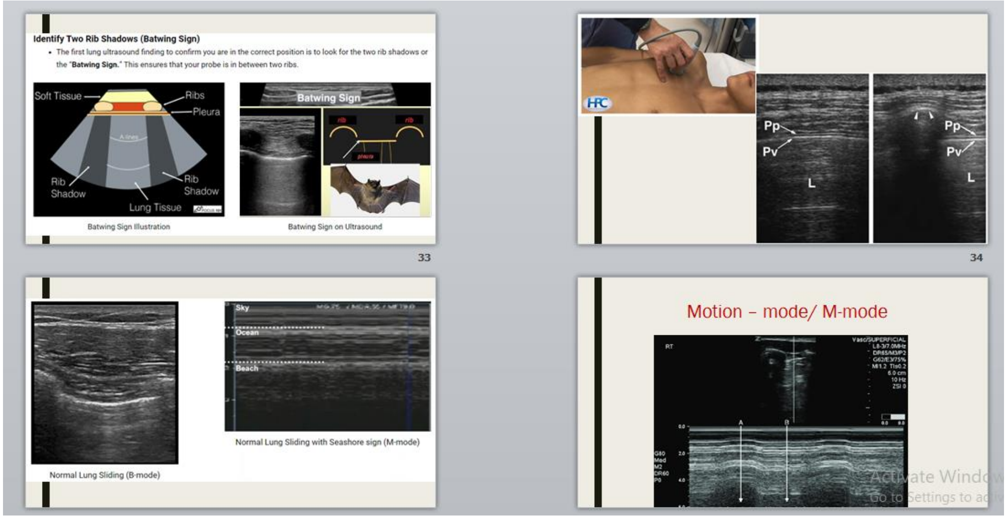
Identify Two Rib Shadows (Batwing Sign)
The first lung ultrasound finding to confirm you are in the correct position is to look for the two rib shadows or the "Batwing Sign." This ensures that your probe is in between two ribs.
Soft Tissue
Ribs
Pleura
Rib Shadow
Rib Shadow
Lung Tissue
Batwing Sign
Batwing Sign Illustration
Batwing Sign on Ultrasound
33
Pp
Pv
L
Pp
Pv
L
34
Sky
Ocean
Beach
Normal Lung Sliding (B-mode)
Normal Lung Sliding with Seashore sign (M-mode)
Motion – mode/ M-mode

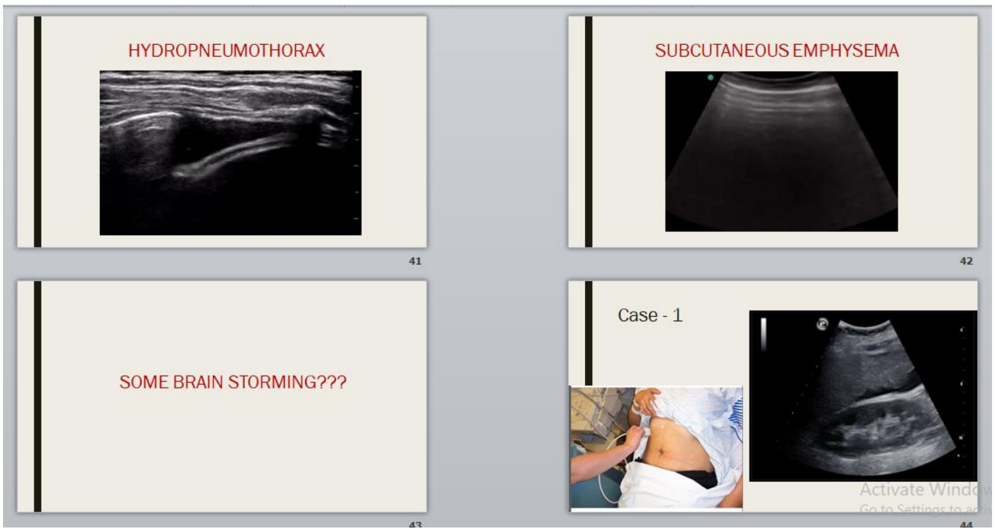
HYDROPNEUMOTHORAX
41
SUBCUTANEOUS EMPHYSEMA
42
SOME BRAIN STORMING???
Case - 1

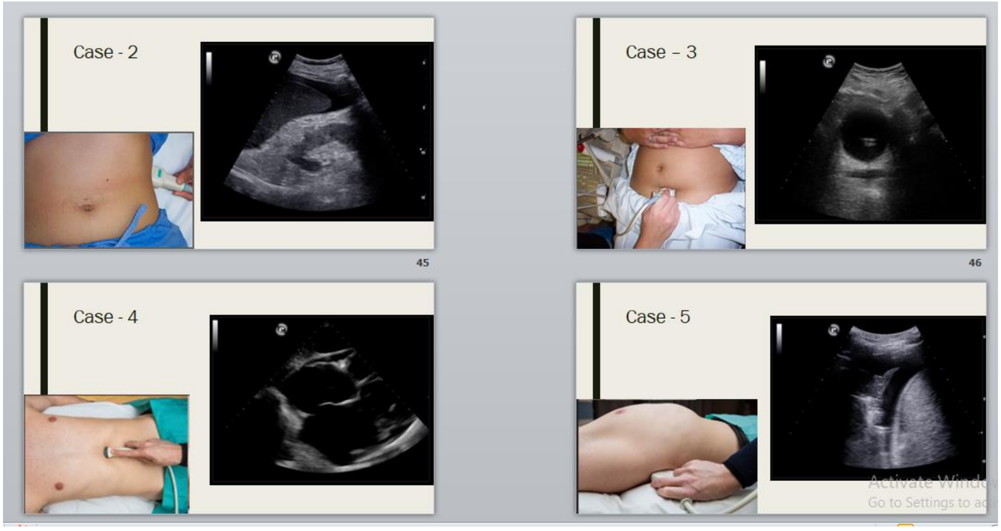
Case - 2
45
Case – 3
46
Case - 4
Case - 5

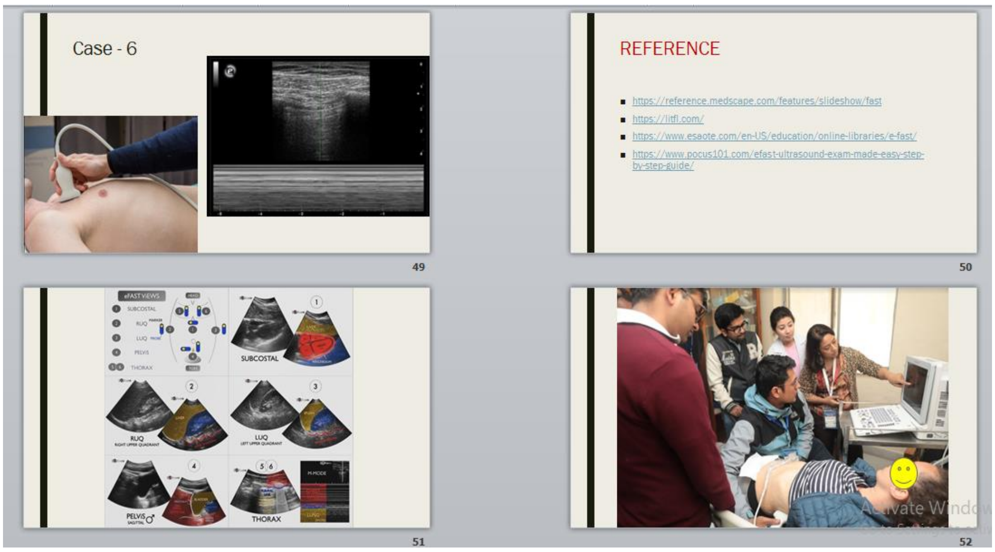
Case - 6
49
REFERENCE
https://reference.medscape.com/features/slideshow/fast
https://litfl.com/
https://www.esaote.com/en-US/education/online-libraries/e-fast/
https://www.pocus101.com/efast-ultrasound-exam-made-easy-step-by-step-guide/
50
SUBCOSTAL
RUQ
LUQ
PELVIS
THORAX
51
52
